# Actigraphic Measurement of the Upper Limbs for the Prediction of Ischemic Stroke Prognosis: An Observational Study

**DOI:** 10.3390/s21072479

**Published:** 2021-04-02

**Authors:** Giuseppe Reale, Silvia Giovannini, Chiara Iacovelli, Stefano Filippo Castiglia, Pietro Picerno, Aurelia Zauli, Marco Rabuffetti, Maurizio Ferrarin, Giulio Maccauro, Pietro Caliandro

**Affiliations:** 1 Department of Geriatrics, Neurosciences and Orthopedics, Università Cattolica del Sacro Cuore, L. Go F. Vito, 1-00168 Rome, Italy; giuseppe.reale@policlinicogemelli.it (G.R.); aureliazauli@gmail.com (A.Z.); giulio.maccauro@policlinicogemelli.it (G.M.); 2 Unità Operativa Complessa Neuroriabilitazione ad Alta Intensità, Largo A. Gemelli, Fondazione Policlinico Universitario A. Gemelli IRCCS, 8-00168 Rome, Italy; 3 Unità Operativa Complessa Medicina Fisica e Riabilitazione, Largo A. Gemelli, Fondazione Policlinico Universitario A. Gemelli IRCCS, 8-00168 Rome, Italy; chiara.iacovelli@policlinicogemelli.it; 4 Department of Medical and Surgical Sciences and Biotechnologies, Sapienza University of Rome, Polo Pontino, Viale XXIV Maggio, 7-04100 Latina, Italy; stefanofilippo.castiglia@uniroma1.it; 5 SMART Engineering Solutions & Technologies Research Center, Università Telematica “e-Campus”, Via Isimbardi, 10-22060 Novedrate, Italy; pietro.picerno@uniecampus.it; 6 Biomedical Technology Department, IRCCS Fondazione Don Carlo Gnocchi, Via Capecelatro, 66-20148 Milan, Italy; mrabuffetti@dongnocchi.it (M.R.); mferrarin@dongnocchi.it (M.F.); 7 Unità Operativa Neurologia, Largo A, Fondazione Policlinico Universitario A. Gemelli IRCCS, Gemelli, 8-00168 Rome, Italy; pietro.caliandro@policlinicogemelli.it

**Keywords:** cerebrovascular diseases, ischemic stroke, prognosis, actigraphy, inertial sensors

## Abstract

Background: It is often challenging to formulate a reliable prognosis for patients with acute ischemic stroke. The most accepted prognostic factors may not be sufficient to predict the recovery process. In this view, describing the evolution of motor deficits over time via sensors might be useful for strengthening the prognostic model. Our aim was to assess whether an actigraphic-based parameter (Asymmetry Rate Index for the 24 h period (AR2_24 h)) obtained in the acute stroke phase could be a predictor of a 90 d prognosis. Methods: In this observational study, we recorded and analyzed the 24 h upper limb movement asymmetry of 20 consecutive patients with acute ischemic stroke during their stay in a stroke unit. We recorded the motor activity of both arms using two programmable actigraphic systems positioned on patients’ wrists. We clinically evaluated the stroke patients by NIHSS in the acute phase and then assessed them across 90 days using the modified Rankin Scale (mRS). Results: We found that the AR2_24 h parameter positively correlates with the 90 d mRS (r = 0.69, *p* < 0.001). Moreover, we found that an AR2_24 h > 32% predicts a poorer outcome (90 d mRS > 2), with sensitivity = 100% and specificity = 89%. Conclusions: Sensor-based parameters might provide useful information for predicting ischemic stroke prognosis in the acute phase.

## 1. Introduction

Stroke is the second-leading cause of death and the third-leading cause of disability worldwide [1]. Although mortality has decreased in past decades, the global burden of stroke (per year incidence, disability-adjusted life-years) is still great and growing, posing a significant challenge to national health systems, who will have to face this challenge in future years [2]. Moreover, during the acute and subacute phase, it is often difficult to formulate a reliable prognosis in terms of 90 d disability. In daily clinical practice, patients with strokes of a similar clinical severity and risk profile can have very different clinical outcomes, even when receiving the same acute treatment. This might have a considerable impact on therapeutic choices and resource allocation, such as rehabilitation programs. We know that clinical severity is the strongest predictor of short-and long-term outcomes after stroke, whereas age, infarct volume and location, etiology, revascularization treatment and comorbidities are additional predictors of outcomes [3,4,5,6,7,8,9]. Some clinical tools address the issue of making a prognostic prediction, but their use is not routine in clinical practice, and a gold-standard tool is not yet available [10,11,12]. Therefore, in everyday clinical practice, stroke physicians formulate a prognosis based on the clinical and instrumental information available on a specific patient, often integrating it with their own personal experience. Hence, establishing an accurate prognostic estimate remains a difficult task, with little consensus among physicians, even when stroke experts take into account well-established stroke predictors [13]. In this context, it is crucial to find additional prognostic factors in order to strengthen the accuracy of outcome prediction. Although there is a growing interest in sensors capable of objectively estimating the clinical severity of stroke in the acute set, no sensor-derived parameter that helps both to predict outcomes reliably and to tailor treatments that currently exist [14,15].

In a previous study conducted on 20 acute ischemic stroke patients, we developed an actigraphic parameter obtained with two actigraphic wristwatch-like sensors that measured upper limb movements over a period of 24 h. We called that index the Asymmetry Rate Index for the 24 h period (AR2_24 h) and demonstrated that it was able to detect and measure the asymmetry of spontaneous movements between the paretic and non-paretic arm [15]. Moreover, we found a direct relationship between the degree of asymmetry and both the National Institutes of Health Stroke Scale (NIHSS) total score and the paretic-upper limb sub-score [15].

Gebruers and colleagues conducted a similar study among acute stroke patients, although it was not performed in the very specialized setting of a sub-intensive care unit, such as a stroke unit [16]. They calculated an actigraphic ratio between the affected and unaffected arm as a ratio between the mean motor activities of both arms over 24 h. Gebruers and colleagues measured arm motor activity using the Proportional Integrating Measure (PIM) approach that integrates the signals from the sensors in order to calculate the area under the rectified curve [16]. Actigraphic signals referred to an epoch of 30 min. They calculated the area under the curve (AUC) for both arms over a 24 h period, and the ratio was then calculated by dividing the AUC of the affected arm by the AUC of the unaffected arm. In this way, the authors developed a ratio as a mean value in a 24 h time period. They found that the motor activity of the affected arm predicted the modified Rankin Scale (mRS) at 3 months better than the ratio between the motor activities of each arm [17,18].

Other groups evaluated upper limb movement symmetry in stroke patients and found correlations between the symmetry and the motor impairment [19,20]. The concept of symmetry and asymmetry used in these studies is based on a function of uni-or triaxial accelerations [19,20], and the ratio between upper limb movement is based on the total time of activity over 24 h [21]. Moreover, these studies evaluated non-bedridden patients with both hemorrhagic and ischemic stroke, with certain selective clinical severity standards (patients with no upper limb movement were excluded), and with different time frames from the index event [19,20].

On the other hand, our approach is based on the epoch-based asymmetry and not on the average of the overall asymmetry. In other words, we measured the motor activity of the upper limbs as synchronous values in epochs of 1 min [22]; these values were then scatter-plotted, with values from the right arm on the x-axis and those from the left arm on the y-axis. The values belonging to the bisecant line indicate a complete symmetry between arms; those in the inferior triangle area indicate the epochs in which motor activity of the right arm is higher than the motor activity of the left arm. The superior triangle indicates a prevalence of motor activity in the left arm. We then calculated the first eigenvector of the data cloud, using the best-fitting line as the index of asymmetry of recordings [22]. In this way, our asymmetry index is not a simple ratio between mean values; conversely, it describes the punctual asymmetry between synchronous values in the upper limbs over time. Hence, AR2_24 h summarizes the dynamic and continuous changesinthe ongoing motor recovery. Moreover, our asymmetry index is a function of the standard deviations of each component of the accelerations [15]. In a previous study, we found that the AR2_24 h index, derived from the analysis of the acceleration components, is more accurate in detecting upper limb movement among acute stroke patients, as compared to the index derived from the analysis of acceleration module. This latter method, in contrast to AR2_24 h, is not able to detect forearm pronosupination movements, which are typically present in bedridden patients [15]. Finally, our index was based on the eigenvector of the components, allowing for the simultaneous evaluation of both upper limbs, providing a different concept of symmetry and asymmetry based on the synchronous comparison of epochs [15].

Therefore, it is reasonable to hypothesize that AR2_24 h might describe the stroke clinical trend with greater accuracy than other methods and could be an additional prognostic predictor in the acute phase. An objective, reproducible measure of motor-recovery trends might be of interest for daily clinical practice, especially if it provides a cut-off with strong positive predictive value power that does not need further interpretation.

The mRS is the most commonly-used scale to evaluate the degree of dependence in daily life after stroke (i.e., generally 90 days after the event). The scale goes from 0 to 6, where 0 indcates no symptoms and 6 indicates death. Values between 0 and 2 indicate no or slight disability, but complete independence; values between 3 and 5 indicate moderate or severe disability. Therefore, we used the mRS at three months to evaluate whether AR2_24 h is effective as a prognostic predictor [23,24].

The aims of the present study were: (1) to analyze the correlation between the actigraphic index and the severity of 90 d disability as quantified by the mRS; (2) to establish the ability of the AR2_24 h index to predict 90 d disability preliminarily.

## 2. Materials and Methods

In this prospective observational study, we enrolled 20 consecutive patients. Inclusion criteria were compromised of: acute middle cerebral artery ischemic stroke within 48–72 h, regardless of the side, location, extension of the ischemic lesion or clinical severity. Exclusion criteria were comprised of: previous ischemic stroke, hemorrhagic stroke, diagnosis of epilepsy and/or cognitive impairment. All patients were previously independent in daily life (mRS 0–1) and right-handed [25].

A stroke neurologist, blinded to the aims of the study, clinically evaluated the patients in the acute phase by collecting NIHSS, 24–36 h Alberta Stroke Program Early CT Score (ASPECTS), stroke aetiology data classified by TOAST classification and acute treatment data [3,9,26,27]. The same neurologist examined patients at the 90 d follow-up and assigned the 90 d mRS [28].

Our sample consisted of bedridden patients during their stay in a sub-intensive stroke unit. For each patient, we measured spontaneous upper limb movement over 24 h using two wristwatch-like actigraphic sensors (EZ430-Chronos, Texas Instruments, Dallas, TX, USA) and obtained the AR2_24 h index as previously described [15,22]. We placed the sensors on the wrists of patients (one sensor per wrist) and synchronized them in order to obtain simultaneous recordings of both limbs. The AR2_24 h does not depend on sensor orientation [22] and therefore represents a robust approach to monitor motor performance in complex environments such as stroke units. Moreover, given the watch-like nature of the sensors, all patients easily accepted having to wear them, which is relevant given that they already need to wear many other sensors that monitor vital functions in the setting of a sub-intensive care unit. In brief, AR2_24 h quantifies the asymmetry of movement between upper limbs over a period of 24 h as percentage values. AR2_24 h assumes that a value of 0% indicates perfectly symmetric behavior, positive values indicate a prevalence of right-side motor activity (up to a maximum of 100% if left activity is absent) and negative values indicate a left-side motor activity prevalence. This means that positive values indicate left hemiparesis/plegia, while negative values indicate right hemiparesis/hemiplegia.

We used the SPSS statistics package (ver. 20.0) for statistical analysis, and the MedCalc Statistical Software (ver. 19.2.6) for the sample size calculation. We assessed the normality of distributions according to the Shapiro–Wilk test. We used the Spearman correlation test to evaluate correlations between AR2_24 h and the 90 d mRS. Finally, we calculated the Receiver Operating Characteristic (ROC) curve in order to identify the best AR2_24 h cut-off value that distinguishes mild (90 days mRS ≤ 2) from moderate-severe disability or death (90 days mRS > 2) [29]. For the latter statistical purpose, the mRS was dichotomized into 0–2 and > 2. We set the sample size calculation in order to test the actigraphic index according to a very high discriminative hypothesis (AUC = 0.90), which was needed to further test its ability to improve predictive models through through longituginal studies with larger samples. We calculated a minimum sample size of at least 16 subjects to test the accuracy of the AR2_24 h index to identify subjects with mRS > 2. If the prevalence of mRS > 2 is 38.9% in the general stroke population [30], this perspective adopts a 5% alpha error and a 20% beta error, and it suggests an excellent discriminative ability (AUC = 0.90) and considers a null hypothesis of 0.50 [31].

For the cut-off value, we chose the point of the ROC curve corresponding to the AR2_24 h value that maximizes the sum of sensitivity and specificity. Sensitivity, specificity, positive and negative predictive values (PPV and NPV, respectively), as well as positive and negative likelihood ratios (LR− and LR+, respectively), were calculated for the optimal cut-off point.

Furthermore, we used a Bayesian approach to estimate the probability of a subject to be predicted correctly as having mRS > 2, in order to avoid potential biases due to the small sample size. We transformed the likelihood ratios, which are accuracy measures independent of the prevalence of mRS > 2 in our sample, into post-test probabilities through Fagan’s nomogram [30,32]. To calculate these post-test probabilities, we used the prevalence of mRS values > 2 in the general stroke population as prior probability, instead of the prevalence of this criterion in our sample. We set *p* values at 0.05.

## 3. Results

In our sample, mean age was 69.2 y with SD:10.1; 12 subjects were female; 9 patients underwent revascularization procedures.

Table 1 shows side of hemiparesis, extension of the ischemic lesion according to the ASPECT score, aetiology according to TOAST classification [26,33], 90 d mRS and actigraphic data (absolute values) of the enrolled stroke sample.

**Figure 1:**
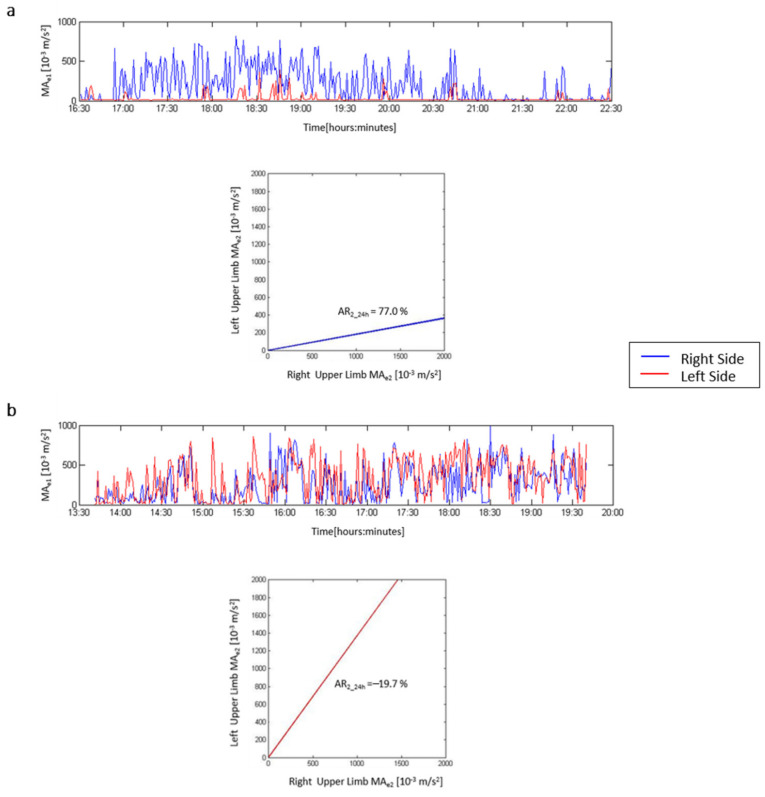
Actigraphy results in two paradigmatic patients. (**a**) Severe left hemiparesis. In the upper plot, the blue line refers to motor activity of the right upper limb over time, whereas the red line refers to the left paretic limb. In the lower graph, the asymmetry index line lies under the bisecant line and indicates a prevalence of right upper limb movements. (**b**) Mild right hemiparesis. In the upper plot, the red line refers to motor activity of the left upper limb over time, whereas the blue refers to the right paretic limb. The asymmetry index line lies above the bisecant line and indicates a slight prevalence of left upper limb movements.

Figure 1 illustrates the motor activity profiles of two different patients; the blue profile refers to the movement of the right wrist, whereas the red profile refers to the left wrist. The patient on the left side (Figure 1a) has a severe left hemiparesis with an AR2_24 h index indicating an important asymmetry and right motor prevalence. The patient on the right side (Figure 1b) illustrates the actigraphic findings in an individual with right mild hemiparesis. In this case, the AR2_24 h index indicates a slight asymmetry, with the motor activity of the left upper limb being greater than that of the right limb.

**Figure 2:**
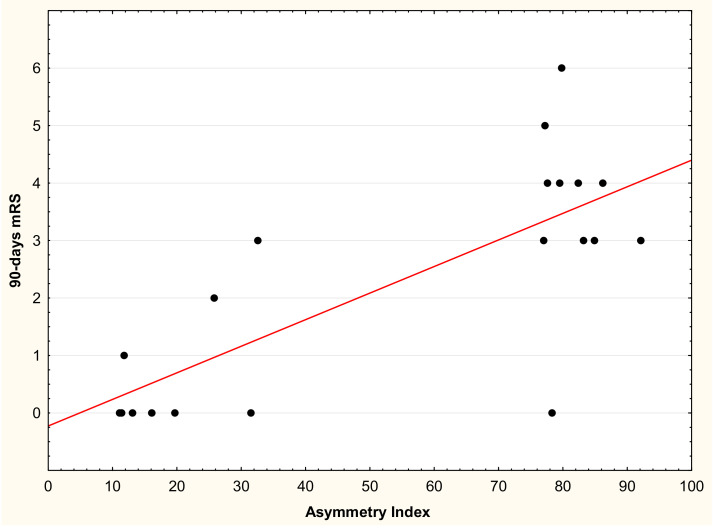
Correlation between the absolute value of the Asymmetry Index and the 90 d modified Rankin Scale (mRS).

We found a positive correlation between AR2_24h and the 90 d mRS (r = 0.69, *p* < 0.001): Low asymmetry index values are associated with low mRS scores, whereas high asymmetry index values are associated with high mRS scores (Figure 2).

**Figure 3:**
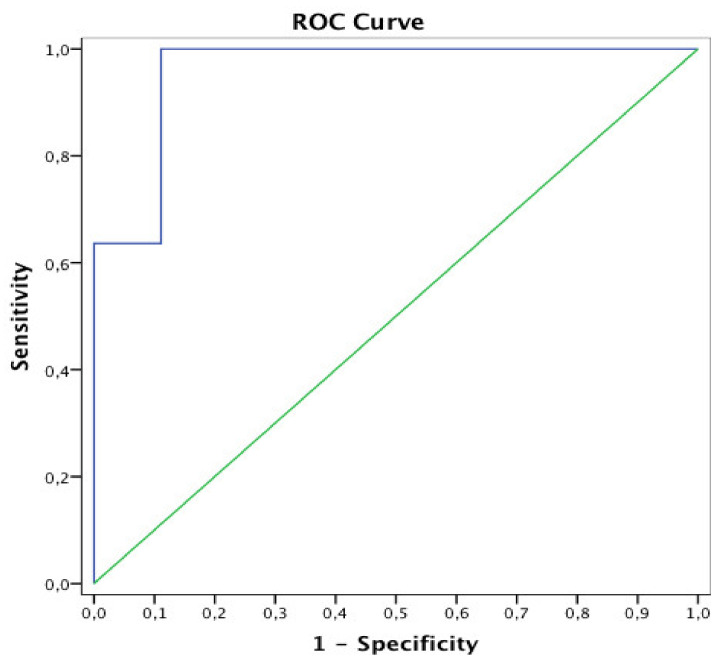
ROC curve for AR2_24 h index predicting 90 d disability (mRS > 2). AUC = 0.96.

We then obtained the ROC curve (Figure 3) and found that the best cut-off value for the asymmetry index able to predict 90 d mRS is 32%: when AR2_24 h > 32%, the asymmetry index predicts a mRS > 2, with sensitivity = 100% and specificity = 89%, and the AUC = 0.96, *p* = 0.001. The PPV of AR2_24 h > 32% in predicting 90 d mRS > 2 was 92%, with NPV = 100%. LR+ was 9.09, and LR− was 0.00. We calculated the post-test probabilities through Fagan’s nomogram, and we found that the accuracy of AR2_24 h > 32% to correctly predict moderate-severe disability at 90 days (PPV) was 85% instead of 92%.

## 4. Discussion

The prognostic evaluation of disability after a stroke is a challenging issue [9,28,34]. Many clinical scales have been developed to predict acute stroke outcomes [10,11,12]. These prognostic scores have advantages and limitations, and not all are routinely included in daily clinical practice for a variety of reasons. First of all, no study has established whether any prognostic score is more reliable than another. Nevertheless, comparing the results of different studies might be misleading, since the tools that were used evaluated different outcomes at different times after the qualifying event [11,12]. Moreover, other factors limit a given method’s routine use, such as feasibility in everyday clinical practice and the relevance of the predicted outcome to a specific clinical context [35,36]. Put together, these issues explain why there is not yet a gold standard prognostic tool, and many researchers struggle to identify new predictors able to improve prognostic models, which should combine clinical and instrumental information. In this context, an easy technology such as actigraphy could implement the multi-parametric monitoring equipment of stroke units. In the present study, we evaluated whether the AR2_24 h index correlates with mRS and found that the index not only correlates with the 90 d prognosis, but also has a cut-off value (AR2_24 h > 32%) that predicts, with high accuracy, a 90 d mRS > 2. Furthermore, we also demonstrated in a previous study that AR2_24 h correlates not only with the paretic upper limb NIHSS sub-score, but also with global stroke severity, as measured by the global NIHSS score [15]. Moreover, recent evidence suggests that the NIHSS score correlates with the 90 d mRS [37], and this presumably explains why we found a positive correlation between the AR2_24 h and the 90 d mRS.

Although we set the sample size calculation according to the hypothesis that the actigraphic index had a very high discriminative ability (AUC = 0.90) in predicting the mRS, the main limitation of our study is the small sample size. This theoretically could have led to a misinterpretation of the diagnostic accuracy of AR2_24 h. In order to interpret the results with greater external validity, we adopted a Bayesian approach and calculated the post-test probability through Fagan’s nomogram [30,32,38]. Even when applying this statistical correction, the efficacy of the asymmetry index was confirmed. Indeed, if we instead used the prevalence of subjects with mRS > 2 in our sample as prior probability, we would have obtained a 92% positive post-test probability. By using the prevalence of subjects with mRS > 2 in the general stroke population, we found a more conservative 85% post-test probability, which is likely the effective probability for detecting a subject with poorer prognosis through the identified cut-off. Furthermore, given that a value of 32% for the AR2_24 h is higher than the highest value in a sample of bedridden subjects with orthopedic conditions [15], it is conceivable that the identified cut-off might truly reflect 90 d disability due to stroke, rather than merely to bed-constraint.

The relationship between the AR2_24 h index and the 90 d mRS appears to be defined by the highest and the lowest values; nevertheless, we do not think that this distribution is due to the small sample size. Indeed, the bimodal distribution of mRS values, defined by the higher prevalence of extreme values, is described in the general stroke population [30]; thus, a bimodal distribution of AR2_24 h values, reflective of the prevalence of disability levels in the general stroke population, was expected.

Regardless, the results of this study should be considered as a proof-of-concept aim to understand the usefulness of the AR2_24 h as an early biomarker of disability, and further longitudinal studies should be conducted to confirm the present outcomes.

These findings support our hypothesis that the continuous quantitative measurement of spontaneous movement might provide additional information when formulating a 90 d prognosis. While standard prognostic factors, such as onset or post-revascularization stroke severity, lesion size, age and comorbidities provide a static frame of the patient in the acute stage, the sensor-based parameters probably better encompass the ongoing dynamic recovery process over 24 h. This justifies further multicentric studies to explore whether this cut-off value is able to strengthen the prognostic accuracy of stroke physicians in a multivariable model, together with classic clinical and imaging-based outcome predictors. If confirmed, this might provide new evidence on the possible routine use of wearable sensors in stroke units in order to monitor stroke recovery, with subsequent valuable information for clinical trend and prognosis.

## 5. Conclusions

Formulating a reliable ischemic stroke prognosis in the acute phase is still challenging, even when all the most-accepted predictors are available, and experienced stroke neurologists make the prediction. Not only are the predictions often wrong, but there is often great interrater variability between physicians. In this regard, objective sensor-based parameters that detail the dynamic evolution of motor recovery, while also providing strong cut-off values that predict good vs. poor outcomes, might be a stimulating add-on to the prognostic model.

## Figures and Tables

**Table 1:** Population clinical and actigraphic characteristics.

Code	Hemiparesis	ASPECTS	Aetiology	Comorbidities	mRS	AR_2_24 h_ Index *
1	L	4	LAA	HT, D	2	25.8%
2	R	10	LAA	HT	0	31.5%
3	R	8	CE	HT, AF	0	19.7%
4	R	9	SVO	HT	0	13.1%
5	R	5	SUAe	HT, CHD, COPD	1	78.3%
6	R	8	LAA	HT	3	83.2%
7	L	7	SUAe	HT	3	32.6%
8	L	4	LAA	HT	3	92.1%
9	L	9	SVO	HT, COPD, CHD	4	82.4%
10	L	6	CE	HT, CHD, HF, AF	6	79.8%
11	R	8	LAA	HT	0	16.1%
12	R	9	SUAe	HT	0	11.4%
13	L	6	SUAe	none	3	84.9%
14	L	8	SUAe	HT	1	11.8%
15	L	5	CE	HT, COPD, AF	5	77.2%
16	R	10	CE	HT, AF	4	79.5%
17	L	5	LAA	none	4	77.6%
18	L	8	CE	HT, AF	3	77.0%
19	R	10	SVO	HT, D	0	11.1%
20	L	6	LAA	HT	4	86.2%

* The last column reports absolute values of Asymmetry indices. Abbreviations: NIHSS: National Institutes of Health Stroke Scale; ASPECTS (24–36 h): Alberta Stroke Program Early CT Score; L: Left; R: Right; LAA: Large-Artery Occlusion; CE: Cardioembolism; SVO: Small-Vessel Occlusion; SUAe: Stroke of Undetermined Aetiology; HT: Hypertension; D: Diabetes; CHD: Coronary heart disease; COPD: Chronic obstructive pulmonary disease; HF: Heart failure; AF: Atrial fibrillation.

## Data Availability

Data are stored in a password-protected PC located in the Institute of Neurology of Fondazione Policlinico Universitario A. Gemelli IRCCS.
